# Supplementary material for: A predictor-informed multi-subject bayesian approach for dynamic functional connectivity
Source: PLoS One. 2024 May 16;19(5):e0298651. doi: 10.1371/journal.pone.0298651 (PMC11098372; doi:10.1371/journal.pone.0298651)
Supplement: S1 File — (PDF) [file pone.0298651.s001.pdf]

Supporting Information

Network	Abbreviation	Full Name	MNI Coordinates
Default Mode Network	PCC	Posterior Cingulate Cortex	(2, 54, 16)
	L pIPL	Left Posterior Inferior Parietal Lobule	(-46, -72, 28)
	R pIPL	Right Posterior Inferior Parietal Lobule	(50, -64, 26)
	PFC/vACC	Orbitofrontal Cortex/Ventral Anterior Cingulate Cortex	(4, 30, 26)
	dMPFC BA 8	Dorsomedial Prefrontal Cortex Broadmann Area 8	(-14, 54, 34)
	dMPFC BA 9	Dosomedial Prefrontal Cortex Brodmann Area 9	(22, 58, 26)
	L DLPFC	Dorsolateral Prefrotnal Cortex	(-50, 20, 34)
	L PHG	Parahippocampal Gyrus	(-10, -38, -2)
	L ITC	Inferolateral Temporal Cortex	(-60, -20, -18)
Fronto-Parietal Control Network	L aPFC	Left Anterior Prefrontal Cortex	(-36, 56, 10)
	R aPFC	Right Anterior Prefrontal Cortex	(34, 52, 10)
	dACC	Dorsal Anterior Cingulate Cortex	N/A
	L DLPFC	Left Dorsolateral Prefrontal Cortex	N/A
	R DLPFC	Right Dorsolateral Prefrontal Cortex	(46, 14, 42)
	L aINS	Left Anterior Insula	(-30, 20, -2)
	R aINS	Right Anterior Insula	(32, 22, -2)
	L aIPL	Left Anterior Inferior parietal Lobule	(-52, -50, 46)
	R aIPL	Right Anterior Inferior Parietal Lobule	(52, -46, 46)
Dorsal Attention Network	L MT	Left MidThalamus	(-44, -64, -2)
	R MT	Right MidThalamus	(50, -70, -4)
	L FEF	Left Frontal Eye Field	(-24, -8, 50)
	R FEF	Right Frontal Eye Field	(28, -10, 50)
	L SPL	Left Superior Parietal Lobule	(-26, -52, 56)
	R SPL	Right Superior Parietal Lobule	(24, -56, 54)
Salience Network	DAC	Dorsal Anterior Cingulate	(0, -22, 36)
	L aPFC	Left Anterior PFC	(-34, 44, 30)
	R aPFC	Right Anterior PFC	(32, 44, 30)
	L Insula	Left Insula	(-40, 2, 6)
	R Insula	Right Insula	(42, 2, 6)
	L LP	Left Lateral Parietal	(-62, -46, 30)
	R LP	Right Lateral Parietal	(62, -46, 30)
Locus Coeruleus	R LC	Rostral Locus Coeruleus	Probabilistic Atlas
	C LC	Caudal Locus Coeruleus	Probabilistic Atlas

S1 Table. ROIs in the case study along with *apriori* defined networks. List of ROIs employed in the case study along with corresponding MNI stereotaxic space coordinates and their classification in *a priori* defined networks.

S1 Fig illustrates how to to specify the value of the parameter τ_0, by simulating 1,000 undirected graphs from the model. A larger τ_0 is associated with higher expected edge densities *a priori*. Additionally, we find that a $\tau_0 = 1$ gives an expected edge density of approximately 50% while having the largest spread.

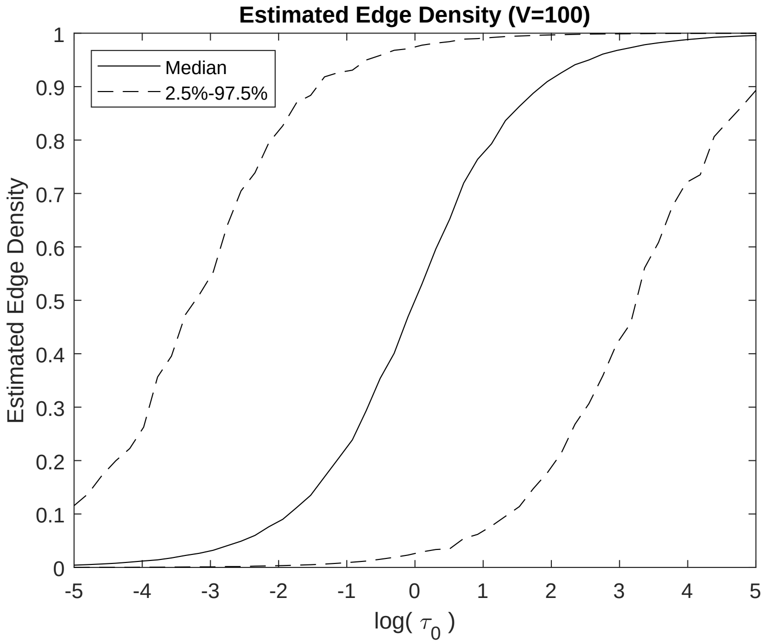

S1 Fig. Sensitivity analysis on the parameter τ_0. For each value of τ_0, 1000 undirected 100×100 graphs are simulated under the graphical horseshoe prior. Shown are the 2.5th, 50th, and 97.5th percentiles of edge density against τ_0. At $\tau_0 = 1$, the expected edge density is approximately 50%, with considerable variability across the sampled graphs.
